# Protocol for halofuginone-mediated metabolic reprogramming of murine T cells via activation of the GCN2 pathway

**DOI:** 10.1016/j.xpro.2025.104173

**Published:** 2025-10-30

**Authors:** Meghan Kates, Michael St. Paul, Pamela S. Ohashi, Samuel D. Saibil

**Affiliations:** 1 Princess Margaret Cancer Center, University Health Network, Toronto, ON M5G 2C1, Canada; 2 Department of Immunology, University of Toronto, Toronto, ON M5S 1C1, Canada

**Keywords:** Cell Biology, Cell isolation, Immunology, Metabolism

## Abstract

Modifying T cell metabolism and activating conserved stress pathways can enhance T cell efficacy in adoptive cell therapy for cancer treatment. Here, we present a protocol to activate the General Control Non-depressible 2 (GCN2)-mediated branch of the integrated stress response (ISR) in murine T cells using the drug halofuginone. We outline the process of isolating CD8^+^ T cells from T cell receptor transgenic mice, activating them with bone-marrow-derived dendritic cells, and subsequently activating GCN2 and the ISR with halofuginone.

For complete details on the use and execution of this protocol, please refer to St. Paul et al.^1^

## Highlights

• Instructions for isolating murine CD8+ T cells
• Steps for co-culturing T cells with bone-marrow-derived dendritic cells
• Guidance for using halofuginone to activate the integrated stress response

## Abstract (editor highlights)

Publisher’s note: Undertaking any experimental protocol requires adherence to local institutional guidelines for laboratory safety and ethics.

## Graphical abstract

**Figure:**
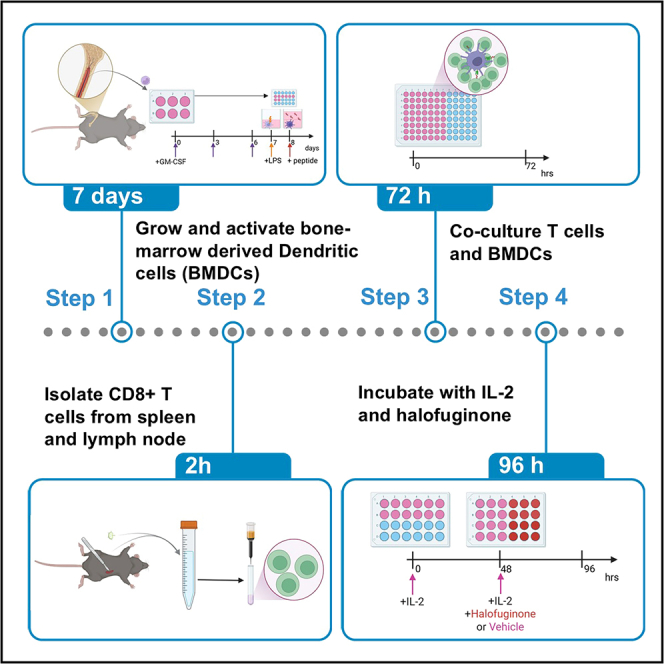

## Before you begin

There is an emerging understanding of the importance of metabolic programming, and adaption to the local metabolic *milieu,* on T cell function, particularly in the context of anti-tumor immunity.^1^^,^^2^^,^^3^^,^^4^^,^^5^ The relative paucity of key metabolites in the tumor microenvironment is believed to activate multiple evolutionary conserved stress pathways. One such pathway is the integrated stress response (ISR), which can be activated in response to amino acid starvation. A scarcity of amino acids leads to accumulation of uncharged transfer RNAs (tRNAs), activating the amino acid starvation sensor general control non-depressible 2 (GCN2).^6^^,^^7^ This kinase then activates the ISR by phosphorylating eukaryotic initiation factor 2 (eIF2α), which inhibits global translation while selectively promoting translation of specific transcripts that help restore amino acid homeostasis. Halofuginone (halo) is a prolyl-tRNA synthetase inhibitor that leads to accumulation of uncharged tRNA, and therefore activation of GCN2.^8^ Halo treatment of murine CD8^+^ T cells leads to upregulation of markers associated with ISR including CD98, increased oxidative metabolism, enhanced interferon (IFN)γ production, and improved ability to clear murine tumors.^1^ Therefore, this represents a promising strategy to improve T cell therapies for cancer.

In this manuscript, we present our optimized protocol for activation of GCN2 in murine T cells using halo. This protocol uses P14 mice that have a transgenic T cell receptor (TCR) that recognizes the gp33 peptide from lymphocytic choriomeningitis virus (LCMV).^9^ However, this methodology is readily adaptable to other transgenic TCR systems recognizing alternative peptides (such as OT-1 cells recognizing ovalbumin peptide) as well as polyclonal T cells.^1^ We describe a step-by-step procedure for isolating and co-culturing P14 T cells with gp33 pulsed bone-marrow derived dendritic cells (DCs). After activation, we outline the steps for induction of the ISR with halo.

### Innovation

With the growing understanding of how metabolic programming affects T cell function, there has been increasing interest in manipulating metabolism to generate better T cells for adoptive cell therapy (ACT). While many approaches have focused on boosting oxidative metabolism to enhance T cell persistence, function and metabolic flexibility *in vivo*, this has been achieved either using glucose-derived carbons or fatty acid oxidation.^5^^,^^10^^,^^11^^,^^12^^,^^13^^,^^14^ However, activation of GCN2 with halo drives oxidative metabolism through autophagy.^1^ This provides either a tool to study metabolism,^15^ or a metabolic strategy with an alternative carbon source for T cells in ACT. Additionally, halo treatment of T cells enhances IFNγ expression and other features that improve anti-tumor activity.

Many strategies exist to activate the GCN2-dependent branch of the ISR,^16^ but halo has attracted particular interest in oncology for its cancer-killing effects across multiple cancer models, which is currently in evaluation in clinical trials.^17^ Despite this interest in halo for cancer therapy, its effects on immune cells remain less explored. Halo has been previously shown to inhibit differentiation of CD4+ T helper 17 (Th17) cells^18^ and proliferation of bulk splenocytes in response to anti-CD3 treatment.^19^ However, the protocol reported here is the first to detail the effects of halo on CD8+ T cells and characterize the use of these cells for ACT.^1^ Additional strengths of this protocol include its adaptability to broad downstream analyses, its reproducibility in other antigen-specific systems and its ability to be applied to polyclonal and human T cells.

### Institutional permissions

All mouse experiments for this protocol were conducted at the Ontario Cancer Institute animal facility. Experiments were conducted according to institutional guidelines with approval of the Ontario Cancer Institute Animal Ethics Committee, Research Ethics Board approval number 929. Before following these procedures, similar approval must be obtained from the researcher’s own institution according to guidelines from the corresponding animal welfare committee.

**Figure 1:**
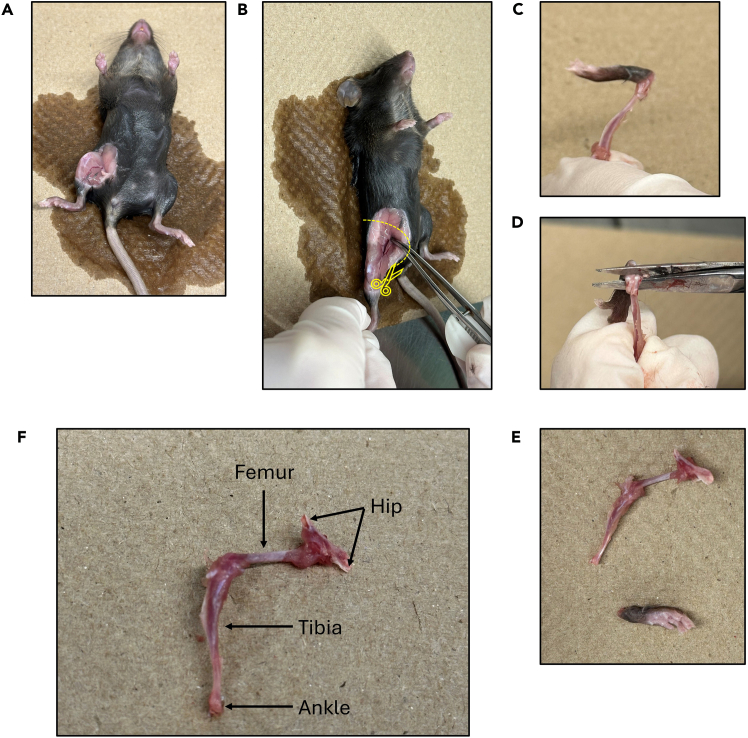
Representative images illustrating key steps of the harvest of the tibia and femur for bone-marrow derived dendritic cell generation (A) Initial incision exposing the femur and hip. (B) Tweezers are placed at the juncture of the hip. Cut following the yellow dotted line to ensure that incisions are made above the hip joint and not into the femur. (C) Bend the paw forward to identify the ankle joint. (D) Insert scissors into the inter-joint space and cut connecting tissues to remove the paw. (E) Illustration of the separated components. (F) After cutting and cleaning, the indicated bones should be visible with the only bone marrow exposed being in the hip.

**Figure 2:**
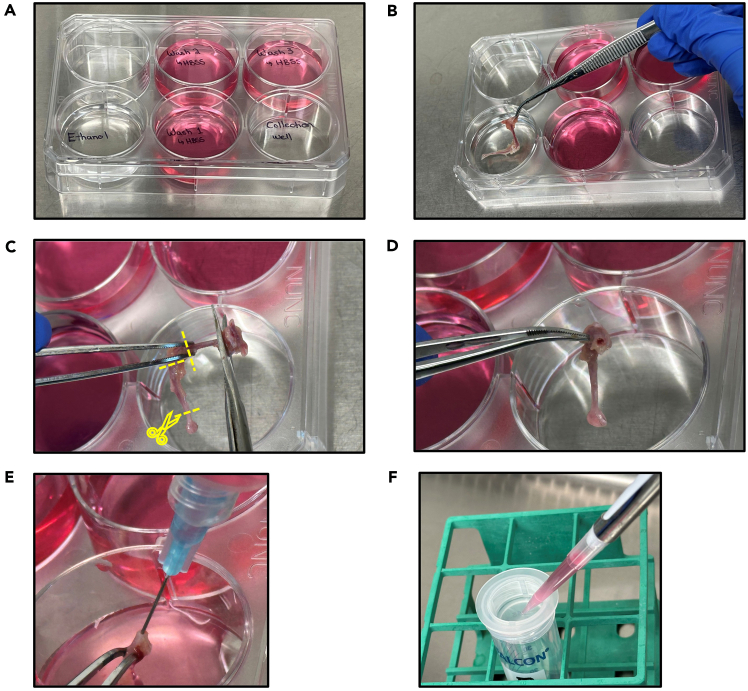
Set-up and process for removing bone marrow from the tibia and femur (A) Set up a 6 well plate accordingly with 1 well of 70% ethanol and 3 wells of HBSS for washing. (B) Transfer the tibia and femur to ethanol. (C) Cut the tibia and femur on both ends along the dotted yellow lines to expose the bone marrow. (D) Exposed bone marrow. (E) Rinse the bone marrow out using a 10 mL syringe filled with HBSS. Let the liquid fall into an empty well of the 6-well plate. (F) Strain the homogenous cell solution through a 70 μm filter on top of a 50 mL tube.

### Media preparation

**Timing: 15 min**

1. Prepare complete RPMI media as described in the materials and equipment section for culture of bone-marrow derived dendritic cells.
2. Prepare complete IMDM media as described in the materials and equipment section for culture of T cells.

### Bone-marrow-derived dendritic cell generation

**Timing: 30 min**

***Note:*** Generation of bone-marrow derived dendritic cells (BMDCs) has also been well described elsewhere.^20^^,^^21^

3. In a biosafety cabinet, set up a 15 mL tube with 5 mL Hank’s balanced salt solution (HBSS).

***Note:*** Ensure that all reagents and samples are kept on ice for the duration of the procedures.

4. Euthanize a female C57BL/6 mouse using inhaled anesthetic (here, 5% isoflurane (volume/volume) was delivered in oxygen at 1 L/min via anesthetic machine, followed by cervical dislocation).

***Note:*** This protocol can use either female or male mice. However, it is important to recognize that the BMDCs generated from the C57BL/6 mouse will later be co-cultured with T cells from a P14 mouse. To eliminate any possible interactions between cells from mice of different sexes, choose to use either both female or both male mice to make BMDCs and T cells.

***Note:*** Euthanization should be performed according to approved institutional protocols.

5. Place the mouse abdomen side up and spray the hind leg with 70% ethanol.
  a. Using scissors, cut away skin to expose the femur and hip (Figure 1A).
  b. Sever the leg with scissors above the hip joints (Figure 1B).
    **CRITICAL:** Avoid cutting into the femur. Do not expose the bone marrow. If done correctly, the only exposed bone marrow should be from the cut edges of the hip bones, which will not be used in the following protocol (Figure 1F).
  c. Remove the fur, muscle and fat from the femur and tibia.
  d. Remove the paw by bending it forward to identify the ankle joint. Insert scissors into the inter-joint space and cut the attaching tissue and tendons. Do not cut bone (Figures 1C–1E).
  e. Place the cleaned bones (Figure 1F) in the 15 mL tube previously set up with HBSS.
6. Transfer 6 mL of HBSS to 3 wells of a 6-well plate. Transfer 6 mL of 70% ethanol to another well of the same 6-well plate (Figure 2A).
7. Using sterile tweezers, transfer the femur and tibia to the ethanol for 1–2 minutes to clean the bones (Figure 2B).

**CRITICAL:** Incubation of the bones in 70% ethanol is required to ensure sterility before exposing the bone marrow. However, if a cut has been mistakenly made, exposing the bone marrow, or if the incubation period runs too long, exposure of the bone marrow to ethanol can occur, leading to bone marrow alterations and damage.

8. Using sterile tweezers, transfer the femur and tibia sequentially to each of the 3 wells with HBSS to dilute out any residual ethanol.
9. Fill a 10 mL syringe with approximately 7 mL HBSS and attach a 25-gauge needle.
10. Using sterile scissors, cut the tibia and femur on both ends to expose the bone marrow (Figures 2C and 2D).
11. Rinse the bone marrow out using the syringe, letting the liquid fall into an empty well of the 6 well plate (Figure 2E).

***Note:*** Flushing the bones with the syringe will collect between 10–20 × 10^6^ cells (not including red blood cells) which is more than sufficient for the downstream experiment.

12. Gently mix the bone marrow solution with a P1000 pipette until it is a uniform single cell suspension (about 10 times).
13. Strain cell mixture through a 70 μm filter into a clean 50 mL tube (Figure 2F).
14. Centrifuge at 450 × *g* for 5 minutes at 4°C.
15. Aspirate supernatant, then resuspend the pellet in 1 mL of complete RPMI.
16. In a tube, mix 450 μL of 1× Ammonium Chloride Potassium (ACK) buffer and 50 μL of cells. Incubate for 2–3 min.

***Note:*** ACK Buffer is used to lyse red blood cells. However, it can cause damage (over-lysis) to other cells if left in solution for too long. To avoid this problem, the cell pellet is resuspended in complete media while a small aliquot is taken for counting that will be exposed to ACK. Therefore, the resulting cell count does not include the red blood cells, which will quickly die off in culture.

17. Count live cells with a hemocytometer or cell counter then resuspend to a final concentration of 10 × 10^7^cells/mL in complete RPMI.
18. In a new 50 mL tube, add 40 μL of granulocyte-macrophage colony-stimulating factor (GM-CSF) stock solution (20 μg/mL) to 20 mL of complete RPMI. Mix.

***Note:*** The final concentration of GM-CSF is 40 ng/mL.

19. Transfer 3 mL of GM-CSF solution to each well of a non-tissue culture treated 6 well plate using a 10 mL pipette.
20. Add 50 μL of cells directly into the middle of each well using a P200 pipette. Drip the cells out slowly in the center of the well with the pipette tip touching the top of the 3 mL volume.
21. Put plate into a 37°C and humidified 5% CO_2_ incubator and incubate for 3 days.

***Note:*** Be careful not to disturb the plate

### Replenish media on day 3

**Timing: 10 min**

22. On day 3 of culture, gently add 3 mL of fresh GM-CSF media (40 ng/mL) on top of each well using a 10 mL pipette.
23. Transfer the plate into a 37°C and humidified 5% CO_2_ incubator for 3 days.

***Note:*** Be careful not to disturb the forming cloud of DCs.

### Replenish media on day 6

**Timing: 10 min**

24. On day 6, remove 3 mL of media from each well using a 10 mL pipette.
25. Gently add 3 mL of fresh GM-CSF media (40 ng/mL) on top of each well using a 10 mL pipette.
26. Transfer the plate into a 37°C and humidified 5% CO_2_ incubator for 3 days.

## Key resources table

**Table:** 

REAGENT or RESOURCE	SOURCE	IDENTIFIER
**Antibodies**		

CD98 (clone RL388)	BioLegend	Cat# 128208; RRID: AB_1186107
CD8 (clone 53–6.7)	eBioscience	Cat#48-0081-82; RRID: AB_1272198

**Chemicals, peptides, and recombinant proteins**		

Gp33	New England Peptide	Custom
Halofuginone (hydrochloride)	Caymen Chemical	Cat #13370
Mouse IL-2	BioLegend	Cat #575402
GM-CSF	PeproTech	Cat #315-03-100UG
LPS from *Eschericihia coli* O111:B4, γ-irradiated, BioXtra, cell culture grade	Sigma-Aldrich	Cat #L4391-10X1MG

**Critical commercial assays**		

CD8a+ T cell isolation kit	Miltenyi	Cat #130-104-075

**Experimental models: Organisms/strains**		

Mouse: P14, 6–8 weeks, female (male can also be used)	Ohashi lab	N/A
Mouse: WT: C57BL/6, 6–8 weeks, female (male can also be used)	The Jackson Laboratory	RRID: IMSR_JAX:000664

**Other**		

RPMI 1640 media	Thermo Fisher Scientific	Cat #: 11875119
IMDM media	Thermo Fisher Scientific	Cat #: 12440061
Fetal bovine serum	Fisher Scientific	Cat #: SH30396.03
Fetal bovine serum, premium	Thermo Fisher Scientific	Cat #: A5670701
Penicillin-streptomycin	Thermo Fisher Scientific	Cat #: 14140122
Β-mercaptoethanol	Sigma-Aldrich	Cat #: M3148-100ML
L-glutamine	Thermo Fisher Scientific	Cat #: 25030081
HBSS with calcium and magnesium	Thermo Fisher Scientific	Cat #: 24020-117
1× PBS	Invitrogen	Cat #: 10010049
MACS separation buffer	Miltenyi	Cat #: 130-091-221
ACK buffer	Thermo Fisher Scientific	Cat #: A1049201
QuadroMACS separator	Miltenyi	Cat #: 130-090-976
MACS MultiStand	Miltenyi	Cat #: 130-042-303
LS column	Miltenyi	Cat #: 130-042-401
Sterile cell strainer	Fisher Scientific	Cat #: 22-363-548
Luer-Lok syringe, 10 mL	BD	Cat #: 302995
Luer-Lok syringe, 3 mL	BD	Cat #: 309657
Needle, 25G	BD	Cat #: 305122
24-well polystyrene clear flat bottom not treated cell culture plate	Corning	Cat #: 351147
6-well clear flat bottom not treated cell multiwell culture plate	Corning	Cat #: 351146

## Materials and equipment

**Table:** Complete RPMI media

Reagent	Final concentration	Amount
RPMI 1640 media	N/A	439.8 mL
Fetal bovine serum	10%	50 mL
L-glutamine	2 mM	5 mL
B-mercaptoethanol	50 μM	200 μL
Penicillin-streptomycin	100 U/mL	5 mL

Fetal bovine serum, L-glutamine and penicillin-streptomycin aliquots are stored at −20°C and must be thawed in a 37°C water bath prior to media preparation. Store final media preparation at 4°C for up to 3 weeks.

**CRITICAL:** Fetal bovine serum used in the RPMI media for preparation of bone-marrow derived dendritic cells should have a low endotoxin level (<10 EU/mL) to prevent premature activation of DCs. The Fetal Bovine Serum, Premium indicated in the key resources table is measured by the vendor to meet the specifications required for growth of dendritic cells.

**Table:** Complete IMDM media

Reagent	Final concentration	Amount
IMDM	N/A	439.8 mL
Fetal bovine serum	10%	50 mL
L-glutamine	2 mM	5 mL
B-mercaptoethanol	50 μM	200 μL
Penicillin-streptomycin	100 U/mL	5 mL

Fetal bovine serum, L-glutamine and penicillin-streptomycin aliquots are stored at −20°C and must be thawed in a 37°C water bath prior to media preparation. Store final media preparation at 4°C for up to 3 weeks.

***Note:*** The endotoxin level in the fetal bovine serum for the IMDM media does not need to be as low as that of the RPMI media. The IMDM media will be used for growth of T cells, which should not be as sensitive to levels of endotoxins, such as lipopolysaccharide. The first fetal bovine serum listed in the key resources table is reported by the vendor as having an endotoxin level ≤25 EU/mL, which is sufficient for this media.

**Table:** Gp33 peptide stock solution

Reagent	Final concentration	Amount
Gp33 peptide	200 μM	–
DMSO	N/A	1 mL (use to initially dissolve peptide)
1× PBS	N/A	Depending on stock peptide amount

Filter with 0.2 μm filter then store stock solution of gp33 peptide at −80°C for up to 12 months.

**Table:** GM-CSF stock solution

Reagent	Final concentration	Amount
GM-CSF	20 μg/mL	100 μg
RPMI	N/A	5 mL

Store stock solution of GM-CSF at −80°C for up to 12 months.

## Step-by-step method details

### Activate DCs with LPS

**Timing: 30 min**

With the plate of BMDCs grown for 6 days in GM-CSF, harvest and treat with lipopolysaccharide (LPS). This step ensures the activation of BMDCs for use in co-culture with T cells.

1. On day 7, use a 10 mL pipette to gently harvest loosely-adherent DCs from each well by pipetting up the well volume then releasing 1–2 times until the loosely-adherent cloud is in solution. Transfer to a clean 50 mL tube.

***Note:*** Gentle pipetting is required to avoid collecting the adherent fraction, which consists of macrophages.^22^ However, it is important to note that the loosely-adherent BMDC fraction is a heterogenous population still comprising macrophages and DCs,^20^ which can be used for this protocol to effectively activate T cells.

2. Centrifuge at 450 × *g* for 5 minutes at 4°C.
3. Remove supernatant then resuspend pellet in 500 μL of complete RPMI.
4. Count cells then resuspend to a final concentration of 2 × 10^6^ cells/mL in complete RPMI.
5. Add LPS to the resuspended cells to a final concentration of 100 ng/mL to activate the BMDCs.
6. Using a P1000 pipette, transfer 1 mL of this solution to each needed well of a non-tissue culture treated 24 well plate.
7. Incubate in a 37°C and humidified 5% CO_2_ incubator for 16 to 24 hours.

**CRITICAL:** Incubating BMDCs with LPS for longer than 24 can lead to progressive cell death.

### Peptide pulse BMDCs

**Timing: 10 min**

Add peptide directly on top of the DC wells set up in the previous step. This step allows for presentation of peptide by the DCs to lead to subsequent activation of T cells.

8. Thaw an aliquot of relevant peptide (in this case gp33) at 18°C–23°C.
9. Pipette 6 mL of RPMI media into a sterile 15 mL tube.
10. Pipette 60 μL of 200 μM stock solution of peptide into the tube of RPMI using a P200 pipette, then gently mix the solution.
11. Transfer 1 mL of peptide solution to each well of BMDCs using a P1000 pipette. To avoid disturbing the BMDCs before harvest, do not mix; the added volume will readily disperse into the existing medium.
12. Transfer the plate of BMDCs to a 37°C and humidified 5% CO_2_ incubator. Incubate for 2 hours.

### Isolation of murine spleen and lymph node

**Timing: 20 min**

This step involves removing the spleen and inguinal lymph nodes from a P14 mouse which can then be used to isolate CD8^+^ T cells.

**Figure 3:**
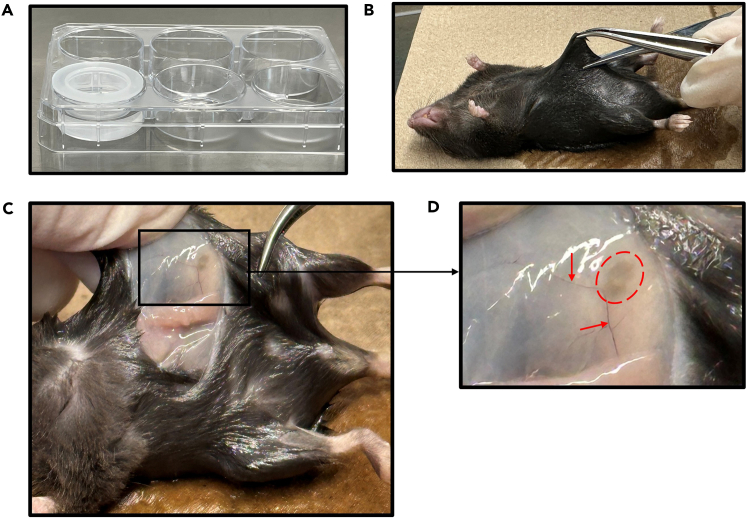
Representative images illustrating key steps for set-up and harvest of the inguinal lymph nodes (A) Set up a 6 well plate with 1× PBS and put a 70 μm filter on top of the liquid so it is sitting in the well. (B) Place the mouse on their back and make an incision along the midline. (C) Peel back the skin on both sides of the incision and identify the inguinal lymph node, located in the black box. (D) Expanded look at the inguinal lymph node which is located at the intersection between two veins (indicated by red arrows). The inguinal lymph node is located in the red dotted circle.

13. In a biosafety cabinet, add approximately 6 mL of sterile, 1× PBS to one well of a 6-well plate.
14. Place a 70 μm filter directly on top of the well so it is sitting on top of the liquid (Figure 3A).

***Note:*** Ensure that all reagents and samples are kept on ice for the duration of the procedures to preserve T cell viability.

15. Euthanize a female P14 mouse using inhaled anesthetic (here, 5% isoflurane (volume/volume) was delivered in oxygen at 1 L/min via anesthetic machine, followed by cervical dislocation).

***Note:*** Euthanization should be performed according to approved institutional protocols.

***Note:*** Use mice that are 6–10 weeks old (after puberty). This protocol can use either female or male mice. However, if a female C57BL/6 mouse has been used to generate BMDCs, then a female P14 mouse should be used (or both male), as noted previously.

**Figure 4:**
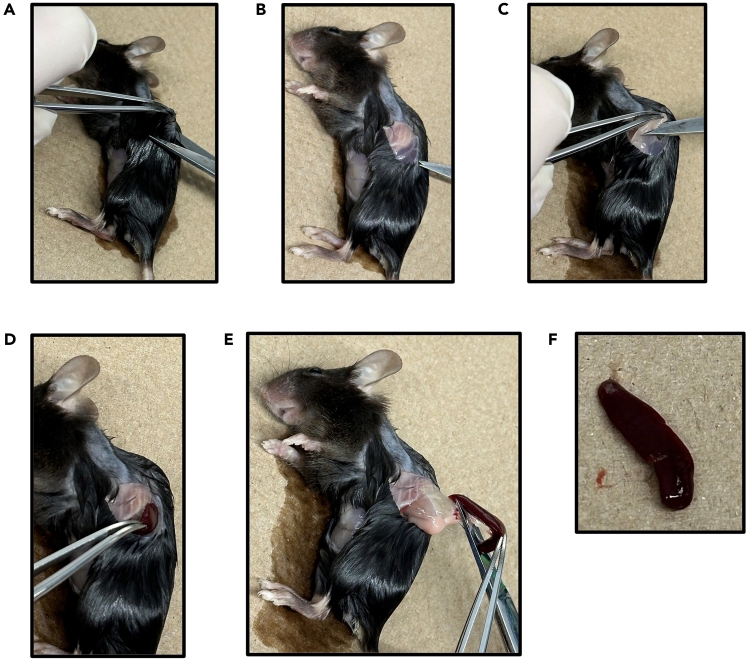
Representative images illustrating key steps for harvest of the spleen (A) Make an incision along the left flank to expose the peritoneum. (B) Spleen is located right below the rib cage as indicated by the edge of the scissors. (C) Make a small incision into the peritoneum to expose the spleen. (D) Gently pull out the spleen to allow it to be (E) gently cut away from the pancreas and surrounding tissue. (F) The spleen is a dark red, kidney bean-shaped organ.

16. Place the mouse on its back and spray with 70% ethanol.
  a. With sterile scissors, make an incision along the midline and peel back the skin on both sides (Figure 3B).
  b. Collect the inguinal lymph node from both flanks using sterile tweezers (Figures 3C and 3D).
    ***Note:*** The inguinal lymph nodes are located attached the skin of the flank at the intersection between two veins (noted by red arrows in Figure 3D). For further guidance on identification of the inguinal lymph nodes, including illustrative diagrams, refer to Harell et al. (2009).^23^
  c. Make an incision along the left flank with scissors to expose the peritoneum (Figures 4A and 4B).
  d. Make a small incision into the peritoneum to expose the spleen (Figure 4C).
  e. Using tweezers, gently pull out the spleen and cut away from the pancreas and surrounding tissue (Figures 4D–4F).
17. Place tissues in the 70 μm filter in the 6-well plate set up in step 14. Ensure that the plate remains on ice to preserve cell viability.

### Isolation of CD8^+^ T cells from spleen and lymph node

**Timing: 1.25 h**

The following steps are used to purify CD8^+^ T cells from murine spleen and lymph nodes to be used in the subsequent co-culture.

18. In a biosafety cabinet, use the plunger of a 3 mL syringe to mash the spleen and lymph node tissue through the 70 μm filter until all tissue besides fat has gone through.
19. Move the filter on top of an open, sterile 50 mL tube.
20. Mix the cell solution in PBS and using a pipette, strain it through the filter into the 50 mL tube.
21. Centrifuge the filtered splenocytes at 450 × *g* for 5 min at 4°C.
22. Remove supernatant.
23. Using the Miltenyi CD8^+^ T cell isolation kit, add 100 μL of CD8 T cell antibody-biotin cocktail. Mix with a pipette.
  a. On top of this, add 400 μL of MACS buffer and mix thoroughly.

***Note:*** Sometimes, clumps will form during this process. These are best to remove at this stage so the resulting cell mixture can be thoroughly mixed.

***Note:*** This procedure uses the Miltenyi isolation kit, but kits from other companies, such as STEMCELL Technologies, can be used instead to isolate CD8^+^ T cells.

24. Incubate the cell mixture for 10 minutes on ice.
25. After incubation, add 200 μL of anti-biotin microbeads from Miltenyi’s CD8^+^ T cell isolation kit. Top up with 300 μL MACS buffer. Mix.
26. Incubate the cell mixture for 15 minutes on ice.
27. After incubation, add MACS buffer to the tube to a total volume of 5 mL.
28. Centrifuge at 450 × *g* for 5 min at 4°C.
29. Set up an LS column on a magnetic rack (such as the QuadroMACS Separator set up on the MACS MultiStand) and suspend over a clean, 15 mL tube.
30. Run 3 mL of MACS buffer through the column.
31. Remove supernatant from cell pellet then resuspend in 1 mL MACS buffer using a P1000 pipette.
32. Once the MACS buffer has run through the column, add the cell suspension to the top of the column.
  a. Once the cell suspension has run through the column, add 3 mL of plain MACS buffer.
33. Using a 5 mL pipette, add 5 mL HBSS to the 15 mL tube (not through the column).
34. Centrifuge at 450 × *g* for 5 min at 4°C.
35. Remove supernatant and resuspend cells in complete IMDM using a P1000 pipette.
36. Count cells.
37. Dilute to a final cell concentration of 1 × 10^6^ cells/mL with complete IMDM.
  a. Keep the isolated T cells on ice until the BMDC collection is completed to maintain cell viability.

### Collect DCs

**Timing: ∼25 min**

These steps outline the harvesting of activated, peptide-pulsed BMDCs for use in activating T cells.

38. After 2 hours of incubation with peptide, harvest DCs by pipetting forcefully with a P1000 pipette. Transfer cell mixture to a sterile 15 mL tube.
39. Centrifuge at 450 × *g* for 5 min at 4°C.
40. Wash 3× in complete IMDM to remove excess free peptide (remove supernatant, resuspend to 10 mL and mix, then centrifuge again 3×).
41. Using a P1000 pipette, resuspend cell pellet in 1 mL of complete IMDM and count cells.
42. Adjust cell concentration to 1 × 10^6^ cells/mL using complete IMDM then dilute 1:10 (1 mL cells at 1 × 10^6^ into 9 mL of IMDM, final concentration 1 × 10^5^ cells/mL).

### Set up co-culture of peptide-pulsed BMDCs and isolated CD8^+^ T cells

**Timing: ∼15 min**

This step involves the preparation of a co-culture of T cells and peptide-pulsed BMDCs to be used for activation of the T cells.

43. Open a sterile 96-well round bottom plate.
44. Pipette 75 μL of T cells (resuspended at 1 × 10^6^ cells/mL) per well using a multi-channel pipette.

***Note:*** This is 75,000 T cells per well

45. Pipette 75 μL of DCs (resuspended at 1 × 10^5^ cells/mL) per well on top of the plated T cells using a multi-channel pipette.

***Note:*** This is 7,500 DCs per well

46. Add 100 μL of complete IMDM to each filled well using a multi-channel pipette.
47. Transfer the plate into a 37°C and humidified 5% CO_2_ incubator. Incubate for 72 hours.

### Harvest activated CD8+ T cells and replate in IL-2

**Timing: ∼20 min**

These steps describe the harvest and re-plating of T cells for further growth before GCN2 activation.

48. Harvest activated T cells using a multi-channel pipette and transfer to a reservoir.
49. Pipette cell suspension into a sterile 15 mL tube using a 10 mL pipette.
50. Centrifuge at 450 × *g* for 5 min at 4°C.
51. Remove supernatant then resuspend cell pellet in complete IMDM using a P1000 pipette.
52. Count cells and resuspend in complete IMDM to a concentration of 1 × 10^6^ cells/mL.
53. Add 1 mL cells to each needed well of a 24-well plate.
54. Resuspend murine IL-2 to a concentration of 20 ng/mL in complete IMDM. (Make enough solution for all plated wells).
  a. Add 1 mL of this IL-2 solution to each plated well. This is a final well concentration of 10 ng/mL IL-2.
55. Put the plate into a 37°C and humidified 5% CO_2_ incubator for 48 hours.

### Addition of halofuginone

**Timing: ∼15 min**

This step details activation of the integrated stress response using the GCN2 agonist, halofuginone.

***Note:*** Halofuginone has acute oral toxicity if swallowed. Perform all work with halofuginone in a biosafety cabinet using standard personal protective equipment. See supplier safety data sheet for further information.

56. Remove the plate from the incubator and mix each well vigorously with a P1000 pipette.
57. Move 1 mL from each well into an empty, adjacent well (split each well in half).
58. Add halofuginone or Vehicle to each well to a final well concentration of 50 ng/mL or equivalent dilution of DMSO.

**CRITICAL:** Halo can be toxic to cells and the optimal dose might need to be titrated depending on the assays to be performed.

***Note:*** Treatment wells should be split between halofuginone treatment and Vehicle control. Halofuginone is dissolved in DMSO.

59. Add 1 mL of IL-2 solution (10 ng/mL) in IMDM on top of each well, regardless of drug treatment.
60. Place the plate into a 37°C and humidified 5% CO_2_ incubator for 48 hours.

***Note:*** At that point the cells are ready for use in downstream analysis including flow cytometry, Seahorse or for use as adoptive cell therapy.

## Expected outcomes

**Figure 5:**
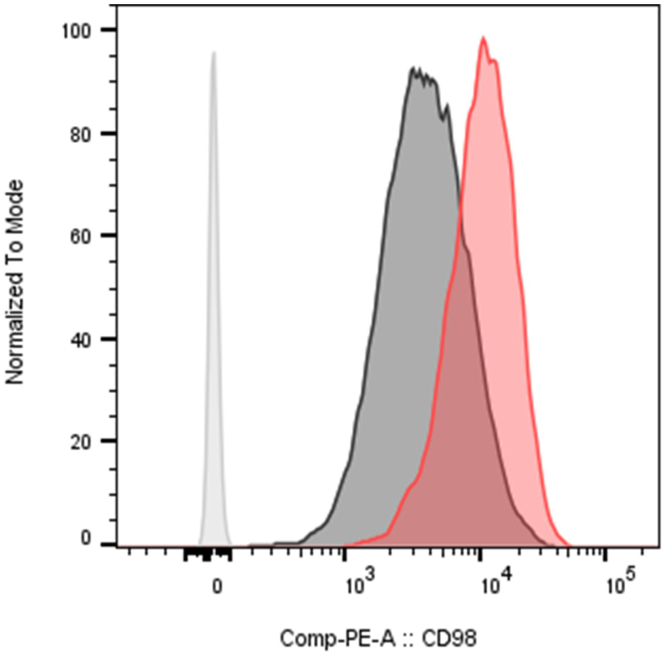
Increased expression of CD98 in halo-treated CD8^+^ T cells (red) compared to vehicle-treated cells (black), n = 1. Fluorescence minus one control is represented in light gray. Data is representative of the results published in St. Paul and Saibil et al., 2024.^1^

**Figure 6:**
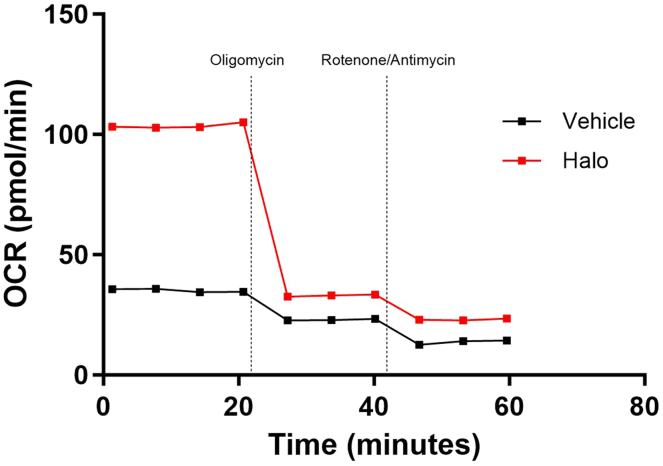
Increased oxygen consumption rate (OCR), measured by Seahorse extracellular flux analyzer, in halo-treated CD8^+^ T cells (red) compared to vehicle-treated cells (black), n=1. Data is representative of the results published in St. Paul and Saibil et al.^1^

**Figure 7:**
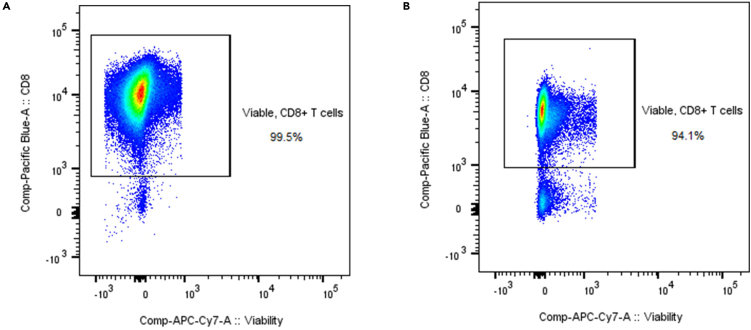
Purity of CD8+ T cells measured by flow cytometry at critical steps of the protocol (A) Purity after Miltenyi CD8^+^ T cell isolation kit. Pre-gated on FSC, SSC, single, viable cells. (B) Purity after 72-hour BMDC-activation of T cells. Pre-gated on FSC, SSC, single, viable cells.

This protocol allows for the generation of activated CD8^+^ T cells that engage the GCN2-mediated ISR. Upon completion of these protocols, halo treated cells should robustly upregulate ISR-associated surface marker, CD98 (Figure 5) and have increased oxidative metabolism (Figure 6). This protocol can be adapted for usage with other T cell subtypes or other immune cells. Additionally, ISR-activated cells can be transferred into mice for adoptive cell therapy treatment of murine tumors. Throughout the process, it is important that sufficient purity and viability of CD8^+^ T cells is achieved. After usage of Miltenyi’s CD8^+^ T cell isolation kit, greater than 90% of cells should be viable and CD8^+^, as measured by flow cytometry (Figure 7A). After 72 hours of co-culture between these cells and activated BMDCs, the BMDCs should have died off in culture, while the resulting cell population should be close to 99% pure for viable, CD8^+^ T cells, as measured by flow cytometry (Figure 7B).

## Limitations

Although treatment of T cells with halo does lead to activation of the ISR, this work was done in nutrient replete media, in which nutrient scarcity does not occur. Therefore, it is unclear to what extent this treatment mimics the phenotype of T cells under nutrient-depleted conditions (such as those in the tumor microenvironment) and should not be used for studying the ISR under starvation. Additionally, T cells in the tumor microenvironment experience many, and dynamic stresses, including but not limited to chronic antigen stimulation, varying nutrient stress, and suppressive signals. Therefore, this protocol would not be representative of the full stress response experienced by tumor-resident T cells. Instead, halo can be used to activate specific aspects of the ISR, including autophagy, and to increase oxidative metabolism to study the effects of this phenotype of T cells in therapeutic settings.

## Troubleshooting

### Problem 1

The halo phenotype can be altered by the composition of the media including differences in nutrient availability or composition of the fetal bovine serum.

### Potential solution

Perform analysis with different lots of fetal bovine serum and different media compositions to determine what provides the most robust and reproducible phenotype.

### Problem 2

Halo can lead to cell death at high concentrations (Addition of halofuginone, step 58).

### Potential solution

**Figure 8:**
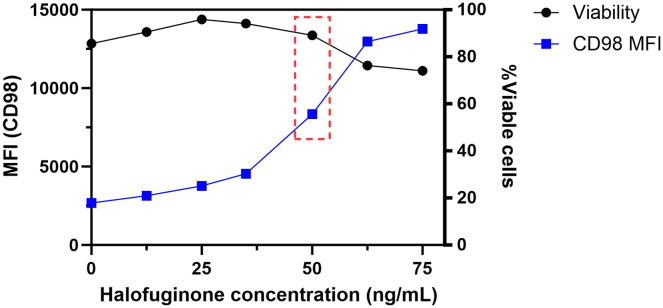
Cell viability (right axis, black), measured by cell counter, and CD98 mean fluorescence intensity (MFI) (left axis, blue), measured by flow cytometry, at varying concentrations of halofuginone The optimal concentration from this experiment, with the greatest increase in CD98 MFI but the lowest reduction in viability, is represented by the dotted red box. Representative data, n = 1.

Titrate the halo concentration to determine the optimal dose range for your cell type and culture conditions. Cell viability and CD98 expression should be used as read-outs to determine the toxicity and efficacy of the treatment, respectively. Choose the concentration with the greatest increase in CD98 with the lowest decrease in viability. For representative data used to choose a halo concentration, see Figure 8.

### Problem 3

Halofuginone can be sensitive to repeated freeze-thaw cycles, which may lead to variability between experiments (Addition of halofuginone, step 58).

### Potential solution

Prepare single-use aliquots that are diluted fresh for each experiment. Do not refreeze once thawed.

### Problem 4

After following the protocol, T cells do not show upregulation of CD98, but do exhibit other functional phenotypes, such as increased oxidative metabolism (OCR) measured by Seahorse.

### Potential solution

Check the flow cytometry antibodies used for CD98 expression. Nearly 100% of activated T cells should express CD98, even in the vehicle-treated group. The shift in CD98 should be assessed by the level of expression or mean fluorescence intensity (MFI).

### Problem 5

After following the protocol, T cells do not have increased oxidative metabolism in Seahorse analysis, despite exhibiting upregulation of CD98.

### Potential solution

Titrate the cell number per well on the Seahorse Analyzer, as T cells can show variability when seeded at low densities. We have used 400,000 activated T cells to achieve confluence in the wells, but this should be optimized for the specific instrument used.

## Resource availability

### Lead contact

Further information and requests for resources and reagents should be directed to and will be fulfilled by the lead contact, Dr. Samuel Saibil (sam.saibil@uhn.ca).

### Technical contact

Technical questions on executing this protocol should be directed to and will be answered by the technical contacts, Meghan Kates (meghan.kates@mail.utoronto.ca) or Dr. Michael St. Paul (michael.stpaul@uhn.ca).

### Materials availability

This study did not generate new unique reagents.

### Data and code availability

RNA sequencing data generated in the published article was deposited at NCBI GEP and are publicly available. The accession number for the RNA sequencing data reported in this paper is "GEO: GSE255244". This study does not report any original code.

## Acknowledgments

The authors thank L. Wybenga-Groot and K. Gill for their technical assistance. This work was supported by a Canadian Institutes of Health Research Foundation Award to P.O. The graphical abstract was created using Biorender.com.

## Author contributions

Writing – original draft, M.K., Designed and supervised the project, M.S.P., S.D.S., and P.O. Assisted with experiments and data analysis, M.K. All authors contributed to the review and editing of the manuscript.

## Declaration of interests

M.S.P., P.O., and S.D.S. hold a patent regarding the use of Halo to enhance the anti-cancer properties of T cells.
